# NSCLC携带EGFR少见突变分析及EGFR-TKIs疗效初步观察

**DOI:** 10.3779/j.issn.1009-3419.2015.08.04

**Published:** 2015-08-20

**Authors:** 雪 杨, 含笑 陈, 弘 张, 建春 段, 彤同 安, 军 赵, 明磊 卓, 梅娜 吴, 洁 王

**Affiliations:** 100142 北京，北京大学肿瘤医院暨北京市肿瘤防治研究所胸部肿瘤内一科，恶性肿瘤发病机制及转化研究教育部重点实验室 Key Laboratory of Carcinogenesis and Translational Research (Ministry of Education), Department of Thoracic Medical Oncology, Peking University Cancer Hospital and Institute, Beijing 100142, China

**Keywords:** 肺肿瘤, EGFR, 少见突变, 靶向治疗, Lung neoplasms, EGFR, Uncommon mutation, Target therapy

## Abstract

**背景与目的:**

表皮生长因子受体（epidermal growth factor receptor, *EGFR*）敏感性突变是EGFR酪氨酸激酶抑制剂（tyrosine kinase inhibitors, TKIs）的有效预测因子。85%-90%敏感性突变发生于19缺失突变及21外显子L858R突变。常见*EGFR*敏感性突变患者EGFR-TKIs治疗的客观缓解率（objective response rate, ORR）和无病进展生存时间（progression-free survival, PFS）显著延长，可分别达70%-80%和9个月-14个月。但EGFR-TKIs对于*EGFR*少见突变（uncommon mutations）的疗效尚不明确。本研究旨在探讨*EGFR*少见突变的临床病理特征及EGFR-TKIs治疗的远近期疗效。

**方法:**

收集2010年4月-2015年4月北京大学肿瘤医院胸部肿瘤内科24例少见*EGFR*突变患者的临床资料，分析少见*EGFR*突变的临床病理特征及与TKIs疗效及PFS之间的关系。

**结果:**

24例携带少见突变的患者中，单突变者15例，双突变者9例。15例单突变中，S768I、L861Q、20外显子插入突变、G719X分别为4例、4例、3例、2例。双突变中以S768I合并G719X最为常见（3/9）。在接受EGFR-TKIs治疗的13例患者中，ORR为46.1%（6/13），疾病控制率（disease control rate, DCR）为76.9%（10/13），中位PFS为7.4个月。

**结论:**

作为特殊类型的*EGFR*突变，*EGFR*少见突变对于一代EGFR-TKIs的敏感性介于*EGFR*敏感性突变和EGFR野生型之间。相对于一代EGFR-TKIs而言，二代EGFR-TKIs可能更适用于*EGFR*少见突变的治疗。。

## Abstract (en)

**Background and objective:**

Epidermal growth factor receptor (*EGFR*) mutations occur more frequently in non-small cell lung cancer (NSCLC) of women, never smokers, Asian population and those with adenocarcinoma. Short in-frame deletion in exon 19 and L858R substitution are the most common mutations, which are closely associated with EGFR tyrosine kinase inhibitors (TKIs) treatment response. However, the therapeutic effects of EGFR-TKIs on NSCLC with uncommon *EGFR* mutation subtypes remain unclear. The aim of this study is to investigate the clinicopathologic feature of uncommon *EGFR* mutations and the outcomes of these patients.

**Methods:**

Twenty-four patients that harbored uncommon *EGFR* mutations were included in this study. Clinicopathologic features of uncommon *EGFR* mutations and the outcomes of these patients were analyzed.

**Results:**

Of the 24 patients, 13 received EGFR-TKIs treatment. The response rate of EGFR-TKIs treatment was 46.1%, and the median progression-free survival (PFS) was 7.4 months. Mutations on S768I and L861Q composed a major part (8 of 24) of uncommon mutations.

**Conclusions:**

Uncommon *EGFR* mutations constituted a unique part of the whole group of *EGFR* mutations. Their composition and sensitivity to EGFR-TKIs were heterogeneous, which requires further assessment in a prospective study.

表皮生长因子受体（epidermal growth factor receptor, *EGFR*）敏感性突变在高加索人群中的发生率为10%-20%，而在亚裔非小细胞肺癌人群（non-small cell lung cancer, NSCLC）中的发生率高达30%-60%^[1-4]^。*EGFR*突变分布不仅与人种相关，在女性、不吸烟、肺腺癌患者中更为常见。针对*EGFR*突变的*EGFR*酪氨酸激酶抑制剂（EGFR-tyrosine kinase inhibitor, EGFR-TKI），由于其客观缓解率（objective response rate, ORR）高达70%-80%，一线PFS可达9个月-14个月，目前已成为*EGFR*突变型NSCLC的一线标准治疗^[1, 3-5]^。

*EGFR*突变主要发生在18号外显子至21号外显子间的区域，而这一区域是酪氨酸激酶的结合区^[6-8]^。*EGFR*敏感性突变中最为常见、与临床疗效最为相关的突变包括19外显子框内缺失突变及21外显子L858R突变，这两类经典突变约占所有*EGFR*突变的85%-90%^[6, 9]^。除了上述两类经典突变外，20外显子T790M突变^[10, 11]^的作用目前也较为明确，即与EGFR-TKIs原发及继发耐药相关。少见突变即除外19缺失突变、L858R、T790M突变以外的所有突变，如E709、G719、S768、L861等位点的氨基酸替换突变^[12, 13]^。

EGFR少见突变由于发生率低，相对常见敏感性突变的研究而言，其临床研究报道寥寥。Beau-Faller等^[14]^对高加索人群中102例少见突变应用一代EGFR-TKIs的疗效进行分析，发现EGFR-TKIs的PFS仅为4个月。而Arrieta等^[15]^研究也获类似结论。在亚洲人群中，Wu等^[16]^对62例少见突变患者一代EGFR-TKIs的疗效进行研究，发现一代EGFR-TKIs的PFS为5个月。除一代EGFR-TKIs研究外，Yang等^[17]^的研究荟萃分析三组有关二代EGFR-TKIs-阿法替尼与一线化疗比较的随机临床研究，对75例携带*EGFR*少见突变患者阿法替尼的治疗疗效进行研究，发现阿法替尼对S768I及L861Q等突变亚型有效，其PFS在8个月-14个月之间。少见突变在不同人种间及不同种类EGFR-TKIs间疗效存在差异。故本研究拟分析就诊于本中心的24例携带*EGFR*少见突变患者的临床病理特征及EGFR-TKIs治疗疗效，为这些突变亚型患者的靶向治疗提供临床数据。

## 1 材料与方法

### 1.1 临床资料

对2010年4月-2015年4月就诊于北京大学肿瘤医院胸部肿瘤内科的24例携带少见*EGFR*突变NSCLC患者的临床资料进行回顾性分析。所有患者均进行了治疗前组织标本*EGFR*基因检测，且为*EGFR*突变型；接受EGFR-TKIs治疗患者定义为至少接受30天吉非替尼、厄洛替尼、埃克替尼或阿法替尼的标准治疗；至少有一个可测量病灶，根据实体瘤的疗效评价标准（Response Evaluation Criteria in Solid Tumors, RECIST）1.1定义的可测量病灶为靶病灶；接受EGFR-TKIs治疗的患者均为Ⅲb期/Ⅳ期。所有标本均以10%福尔马林固定，常规石蜡包埋封存。

### 1.2 ARMS及测序法检测*EGFR*突变

采用扩增阻滞突变系统（amplification refractory mutation system, ARMS）法或测序法分析各样本中*EGFR*基因突变状况（包括18外显子G719S、G719A、G719C、G719X，21外显子L858R、L861Q、L833V、H835L，19外显子缺失突变，20外显子插入突变、T790M、S768I等突变）。

### 1.3 疗效评价

EGFR-TKIs治疗1个月后进行首次计算机断层扫描（computed tomography, CT）复查，根据RECIST 1.1标准进行疗效评价，未进展的患者按美国国立综合癌症网络（National Comprehensive Cancer Network, NCCN）推荐进行CT复查及随访。PFS定义为自EGFR-TKIs治疗开始至出现有客观证据证明疾病进展的时间。随访时间截至2015年5月18日。

### 1.4 统计学方法

采用SPSS 17.0统计软件处理数据。

## 2 结果

### 2.1 少见突变患者的一般临床资料

在24例*EGFR*少见突变NSCLC患者中，男性11例，女性13例。中位年龄62岁（年龄40岁-77岁）。吸烟8例，不吸烟16例。腺癌23例，鳞癌1例。根据2009年国际抗癌联盟（Union for International Cancer Control, UICC）/美国癌症联合委员会（American Joint Committee on Cancer, AJCC）联合制定的第7版进行肿瘤-淋巴结-转移（tumor node metastasis, TNM）分期，其中Ⅰ期3例，Ⅱ期2例，Ⅲ期6例，Ⅳ期13例。所有患者临床病理特征分布见表 1。

**1:** 入组患者的临床病理特征 Clinicopathologic features of 24 patients with NSCLC

Characteristic	Number	Percentage (%)
Age, years		
Median	62	
Range	40-77	
Gender		
Male	11	46
Female	13	54
Smoking status		
Never	16	67
Ever	8	33
Histology		
Adenocarcinoma	23	96
Squamous carcinoma	1	4
TNM staging		
Ⅰ	3	13
Ⅱ	2	8
Ⅲ	6	25
Ⅳ	13	54
NSCLC: non-small cell lung cancer; TNM: tumor-node-metastasis.		

### 2.2 少见突变类型分析

24例少见突变中单突变者15例，其中S768I单突变4例，L861Q单突变4例，20外显子插入突变3例，G719X单突变2例，G719S单突变1例，L833V单突变1例。

双突变者9例，其中G719X及S768I双突变3例，S768I及V769L双突变1例，S768I及T790M双突变1例，S768I及L861Q双突变1例，L861Q及T790M双突变1例，20外显子Q787Q同义突变合并L858R 1例，L833V及H835L突变1例。突变类型分布见表 2。

**2:** *EGFR*少见突变类型分布 Types of *EGFR* uncommon mutation

No. of patients	*EGFR* mutation	Mutation exon
1	G719S	18
2	G719X	18
4	S768I	20
3	Exon 20 insertion	20
1	L833V	21
4	L861Q	21
1	L833V+H835L	21
3	S768I+G719X	18+20
1	S768I+L861Q	18+20
1	S768I+T790M	20
1	S768I+V769L	20
1	L861Q+T790M	20+21
1	Q787Q synonymous mutation+L858R	20+21
EGFR: epidermal growth factor receptor.		

### 2.3 少见突变与EGFR-TKIs疗效相关性分析

对24例少见突变患者的临床病例资料进行回顾性分析，发现13例患者接受EGFR-TKIs治疗（治疗期间未联合化疗或局部放疗）。其中7例为吉非替尼治疗，4例为厄洛替尼治疗，1例为埃克替尼治疗，1例为阿法替尼治疗。EGFR-TKIs为一线治疗的患者9例，二线及以上者4例。其中有4例为术后复发转移，其余9例确诊时均为Ⅳ期。少见突变患者应用EGFR-TKIs的ORR为46.1%（6/13），疾病控制率（disease control rate, DCR）为76.9%（10/13），3例患者在EGFR-TKIs治疗1个月后即出现疾病进展（progressive disease, PD）。截至末次随访时间2015年5月18日，共有10例患者发生疾病进展，中位PFS为7.4个月（范围1.1个月-21.7个月）（图 1）。13例患者临床病理特征及对EGFR-TKIs疗效见表 3。

**3:** 13例患者接受EGFR-TKIs治疗患者的临床病理特征及疗效 Summary of clinical information of patients treated with EGFR-TKIs

Pt ID	Sex	Age (year)	Smoking	Stage	Histology	*EGFR* mutation	TKI response	PD	PFS (mo)
1	M	62	Y	Ⅳ	ADC	S768I+V769L	PR	Y	1.2
2	M	77	N	Ⅳ	ADC	S768I+G719X	PR	Y	8.8
3	M	60	N	Ⅳ	ADC	G719S	SD	Y	13.1
4	M	40	Y	ⅢA→Ⅳ	ADC	S768I+L861Q	SD	Y	3.9
5	M	76	Y	Ⅳ	ADC	Q787Q +L858R	PR	Y	21.7
6	F	49	N	ⅢA→Ⅳ	SCC	L833V	SD	N	NE
7	F	56	N	Ⅳ	ADC	S768I	PR	N	NE
8	F	70	N	Ⅳ	ADC	L861Q	PR	Y	7.4
9	F	63	N	Ⅳ	ADC	L861Q	PD	Y	1.1
10	F	61	N	ⅡA→Ⅳ	ADC	S768I+T790M	SD	Y	9.1
11	M	62	Y	Ⅳ	ADC	Exon 20 ins	PR	N	NE
12	F	59	N	ⅡB→Ⅳ	ADC	Exon 20 ins	PD	Y	1.1
13	F	65	N	Ⅳ	ADC	L833V+H835L	SD	Y	6.7
Pt: patient; M: male; F: female; Y: Yes; N: No; ADC: adenocarcinoma; SCC: squamous cell carcinoma; PR: partial remission; SD: stable disease; NE: not estimatable; TKI: tyrosine kinase inhibitor.									

**1:**
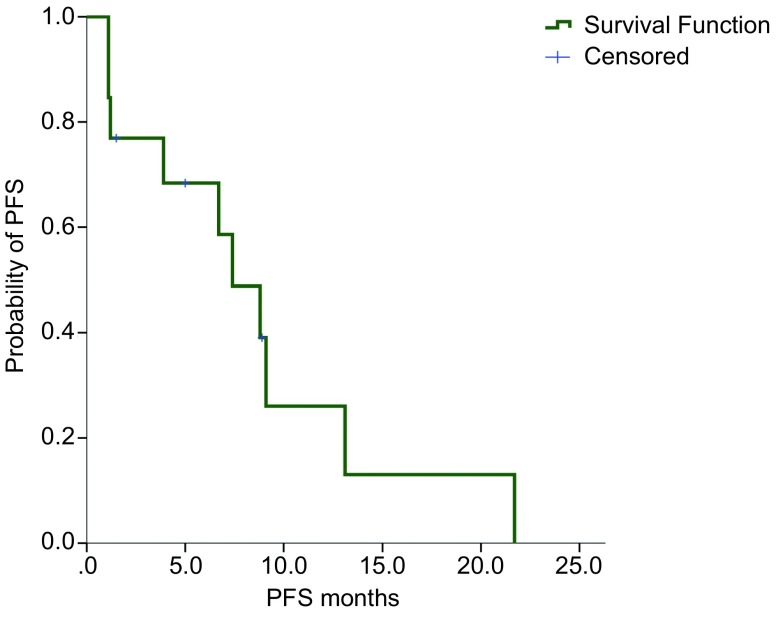
*EGFR*少见突变患者接受EGFR-TKIs的PFS The PFS analysis of patients with EGFR uncommon mutations treated with EGFR-TKIs

值得一提的是，4号患者两次应用EGFR-TKIs。该患者同时存在S768I及L861Q双突变，二线应用吉非替尼最佳疗效为疾病稳定（stable disease, SD），PFS为3.9个月。在三线化疗进展后，四线应用厄洛替尼治疗，最佳疗效为SD，PFS为12.2个月。

## 3 讨论

本研究对24例*EGFR*少见突变的临床病理特征及与EGFR-TKIs疗效相关性进行深入分析，发现*EGFR*少见突变在接受EGFR-TKIs治疗时，ORR为46.1%，DCR为76.9%，中位PFS为7.4个月。*EGFR*少见突变无论是ORR还是PFS，都较经典突变低，但较EGFR野生型患者高。

本研究少见突变位点中最常发生的突变位点为S768I。S768I突变是发生在*EGFR*基因20外显子768号密码子的点突变（G > T）。本研究发现S768I单突变4例，合并其他突变者6例，S768I发生双突变的概率为60%，这与Chen等^[18]^报道在*EGFR*基因G719、S768、T790、L861等区域更易合并双突变的结果部分一致。既往基础研究提示相对于G719S及L861Q而言，吉非替尼或厄洛替尼对于S768I细胞系的IC_50_值更高，提示S768I突变可能对吉非替尼耐药^[19]^。但Masago等^[20]^曾报道过1例S768I单突变患者，二线吉非替尼的PFS长达15个月。本研究中1例S768I单突变患者一线接受阿法替尼治疗，目前已随访5个月，尚未出现疾病进展。另2例S768I分别合并G719X、T790M双突变患者一代EGFR-TKIs的PFS均在9个月左右，而2例S768I分别合并L861Q、V769L双突变患者PFS分别为3.9个月及1.2个月。提示第一代EGFR-TKIs对于部分S768I突变可能有一定疗效，相较一代EGFR-TKIs，二代EGFR-TKIs可能对S768I突变更加有效。

除S768I外，本研究中另一常见的突变位点为21外显子的L861Q。既往研究^[21]^报道L861Q突变占所有*EGFR*突变的2%。目前有关L861Q突变对于EGFR-TKIs药物反应的报道不尽相同。部分研究提示第一代EGFR-TKIs对于L861Q突变完全无效^[22]^，但也有研究提示EGFR-TKIs对L861Q突变部分有效，但敏感性低于L858R及G719S突变^[13]^，而二代EGFR-TKIs^[23]^可较为有效地抑制L861Q突变。本研究中两例接受一代EGFR-TKIs治疗的L861Q突变患者，PFS分别为1.1个月和7.4个月，提示L861Q突变的NSCLC可能是一类异质性较强的肿瘤，临床上一线选择第一代EGFR-TKIs治疗L861Q突变者需慎重。

18外显子的点突变包括第719位点的甘氨酸被丝氨酸、丙氨酸或半胱氨酸(G719S/A/C)所取代。体外研究^[24]^提示G719突变型与ATP亲和力介于野生型EGFR及L858R之间。既往研究^[16]^提示18外显子G719的点突变无论是单突变还是双突变，ORR均可达53.3%，中位PFS为8.1个月。而Chiu等^[25]^报道G719X单突变ORR为36.8%，而G719X合并S768I双突变ORR为50%。本研究中仅有1例G719X合并S768I的双突变患者接受EGFR-TKIs治疗，EGFR-TKIs最佳疗效为部分缓解（partial remission, PR），PFS为8.8个月，接近经典突变（EGFR 19和21）患者接受EGFR-TKIs治疗的疗效及PFS。G719X合并其他突变的双突变较G719X单突变疗效更好的原因可能是与单突变相比，EGFR少见双突变可能对于ATP亲和力更强，对于EGFR-TKIs更敏感，因而EGFR-TKIs治疗具有较高的ORR及较长的PFS。而二代EGFR-TKIs阿法替尼对于G719X的ORR可达78%，中位13.8个月，明显优于一代EGFR-TKIs^[17]^。

20外显子插入突变约占所有*EGFR*突变的1%-10%，与EGFR-TKIs耐药相关。二代EGFR-TKIs临床前数据表明20外显子插入突变的IC_50_值与T790M突变类似，约为*EGFR*敏感突变的100倍。Wu等^[26]^报道2例20外显子插入突变应用第一代EGFR-TKIs的PFS分别为1个月和2.5个月。Yang等^[17]^报道23例接受阿法替尼治疗的20外显子插入突变患者，ORR为8.7%，DCR为65.2%，中位PFS为2.7个月，表明20外显子插入突变对EGFR-TKIs耐药。不过，本研究3例20外显子插入突变的患者，2例接受第一代EGFR-TKIs治疗。其中1例吉非替尼一线治疗PFS为1.1个月，另1例接受厄洛替尼一线治疗1.5个月，最佳疗效达到PR，目前仍在厄洛替尼治疗中。因此，20外显子插入突变是否对所有EGFR-TKIs均无效，抑或有效但维持时间短，而后出现快速进展，对此尚无明确结论，尚需更多的回顾性或前瞻性研究数据证实。

1例患者同时检测到H835L及L833V双突变。根据文献检索结果，目前国内外文献中共有6例L833V及H835L双突变的报道，均为亚裔患者，最长PFS为8.5个月^[27-29]^。本研究中L833V及H835L双突变患者也为亚裔，65岁女性，一线接受吉非替尼治疗，PFS为6.7个月，提示H835L及L833V双突变可归于EGFR-TKIs敏感突变。

1例患者同时检测到L858R及Q787Q双突变。Q787Q突变为20外显子CAG-CAA同义突变，多与其他*EGFR*突变伴随存在。Peng等^[30]^报道Q787Q在少见突变中的发生率约36.3%（8/22）。Kim等^[31]^分析Q787Q单突变患者25例，其中15例患者可进行EGFR-TKIs疗效分析。EGFR-TKIs治疗Q787Q单突变的ORR为13.3%，中位进展时间（time to progression, TTP）为12.9个月。本研究中1例L858R及Q787Q双突变患者PFS为21.7个月，与敏感突变PFS类似。此例患者的PFS可能与L858R这种敏感突变更为相关。

根据文献检索结果，目前国内外文献中共有6例L833V及H835L双突变的报道，均为亚裔患者，最长PFS为8.5个月。本研究中L833V及H835L双突变患者也为亚裔，65岁女性，一线接受吉非替尼治疗，PFS为6.7个月，提示H835L及L833V双突变可归于EGFR-TKIs敏感突变。

4号患者为40岁男性，同时存在S768I及L861Q双突变。该患者两次应用EGFR-TKIs。有趣的是，该患者二线治疗应用吉非替尼，其PFS仅为3.9个月，但经过化疗后，四线应用厄洛替尼PFS可达12.2个月。二线EGFR-TKIs PFS之间差异的原因考虑：①药物空间构象、与酪氨酸激酶亲和力之间的差异。吉非替尼为EGFR-TKIs最低有效剂量，而厄洛替尼为最大耐受剂量，两种药物之间有效含量的差异可能是造成疗效差异的原因之一。故对携带少见突变的患者，选择有效剂量较高的药物抑或是二代不可逆的EGFR-TKIs可能是更为合理的策略；②化疗清除部分对EGFR-TKIs耐药的克隆，存留的克隆对EGFR-TKIs敏感，因此后续应用EGFR-TKIs的PFS较长。

综上所述，*EGFR*少见突变作为特殊类型的*EGFR*突变，包含各种亚型，不同亚型对EGFR-TKIs的敏感性不尽相同。本研究中大部分少见突变的携带患者接受EGFR-TKIs治疗的ORR和PFS较经典的敏感性突变低，但较EGFR野生型患者高。与一代EGFR-TKIs相比，二代EGFR-TKIs可能更适用于*EGFR*少见突变的治疗。

## References

[1] Mok TS, Wu YL, Thongprasert S (2009). Gefitinib or carboplatin-paclitaxel in pulmonary adenocarcinoma. N Engl J Med.

[2] Rosell R, Moran T, Queralt C (2009). Screening for epidermal growth factor receptor mutations in lung cancer. N Engl J Med.

[3] Mitsudomi T, Morita S, Yatabe Y (2010). Gefitinib versus cisplatin plus docetaxel in patients with non-small-cell lung cancer harbouring mutations of the epidermal growth factor receptor (WJTOG3405): an open label, randomised phase 3 trial. Lancet Oncol.

[4] Zhou C, Wu YL, Chen G (2011). Erlotinib versus chemotherapy as first-line treatment for patients with advanced *EGFR* mutation-positive non-small-cell lung cancer (OPTIMAL, CTONG-0802): a multicentre, open-label, randomised, phase 3 study. Lancet Oncol.

[5] Rosell R, Carcereny E, Gervais R (2012). Erlotinib versus standard chemotherapy as first-line treatment for European patients with advanced *EGFR* mutation-positive non-small-cell lung cancer (EURTAC): a multicentre, open-label, randomised phase 3 trial. Lancet Oncol.

[6] Lynch TJ, Bell DW, Sordella R (2004). Activating mutations in the epidermal growth factor receptor underlying responsiveness of non-small-cell lung cancer to gefitinib. N Engl J Med.

[7] Paez JG, Janne PA, Lee JC (2004). *EGFR* mutations in lung cancer: correlation with clinical response to gefitinib therapy. Science.

[8] Gu D, Scaringe WA, Li K (2007). Database of somatic mutations in *EGFR* with analyses revealing indel hotspots but no smoking-associated signature. Hum Mutat.

[9] Sharma SV, Bell DW, Settleman J (2007). Epidermal growth factor receptor mutations in lung cancer. Nat Rev Cancer.

[10] Shih JY, Gow CH, Yang PC (2005). *EGFR* mutation conferring primary resistance to gefitinib in non-small-cell lung cancer. N Engl J Med.

[11] Kosaka T, Yatabe Y, Endoh H (2006). Analysis of epidermal growth factor receptor gene mutation in patients with non-small cell lung cancer and acquired resistance to gefitinib. Clin Cancer Res.

[12] Shigematsu H, Lin L, Takahashi T (2005). Clinical and biological features associated with epidermal growth factor receptor gene mutations in lung cancers. J Natl Cancer Inst.

[13] Pallis AG, Voutsina A, Kalikaki A (2007). 'Classical' but not 'other' mutations of EGFR kinase domain are associated with clinical outcome in gefitinib-treated patients with non-small cell lung cancer. Br J Cancer.

[14] Beau-Faller M, Prim N, Ruppert AM (2014). Rare EGFR exon 18 and exon 20 mutations in non-small-cell lung cancer on 10 117 patients: a multicentre observational study by the French ERMETIC-IFCT network. Ann Oncol.

[15] Arrieta O, Cardona AF, Corrales L (2015). The impact of common and rare *EGFR* mutations in response to EGFR tyrosine kinase inhibitors and platinum-based chemotherapy in patients with non-small cell lung cancer. Lung Cancer.

[16] Wu JY, Yu CJ, Chang YC (2011). Effectiveness of tyrosine kinase inhibitors on "uncommon" epidermal growth factor receptor mutations of unknown clinical significance in non-small cell lung cancer. Clin Cancer Res.

[17] Yang CH, Sequist L, Geater S (2013). Activity of afatinib in uncommon epidermal growth factor receptor (*EGFR*) mutations: findings from three trials of afatinib in *EGFR* mutation-positive lung cancer. World Congress on Lung Cancer(WCLC).

[18] Chen Z, Feng J, Saldivar JS (2008). EGFR somatic doublets in lung cancer are frequent and generally arise from a pair of driver mutations uncommonly seen as singlet mutations: one-third of doublets occur at five pairs of amino acids. Oncogene.

[19] Kancha RK, von Bubnoff N, Peschel C (2009). Functional analysis of epidermal growth factor receptor (*EGFR*) mutations and potential implications for EGFR targeted therapy. Clin Cancer Res.

[20] Masago K, Fujita S, Irisa K (2010). Good clinical response to gefitinib in a non-small cell lung cancer patient harboring a rare somatic epidermal growth factor gene point mutation; codon 768 AGC > ATC in exon 20 (S768I). Jpn J Clin Oncol.

[21] Mitsudomi T, Yatabe Y (2010). Epidermal growth factor receptor in relation to tumor development: *EGFR* gene and cancer. FEBS J.

[22] Hsieh MH, Fang YF, Chang WC (2006). Complex mutation patterns of epidermal growth factor receptor gene associated with variable responses to gefitinib treatment in patients with non-small cell lung cancer. Lung Cancer.

[23] Kancha RK, Peschel C, Duyster J (2011). The epidermal growth factor receptor-L861Q mutation increases kinase activity without leading to enhanced sensitivity toward epidermal growth factor receptor kinase inhibitors. J Thorac Oncol.

[24] Yun CH, Boggon TJ, Li Y (2007). Structures of lung cancer-derived *EGFR* mutants and inhibitor complexes: mechanism of activation and insights into differential inhibitor sensitivity. Cancer Cell.

[25] Chiu CH, Yang CT, Shih JY (2015). Epidermal growth factor receptor tyrosine kinase inhibitor treatment response in advanced lung adenocarcinomas with G719X/L861Q/S768I mutations. J Thorac Oncol.

[26] Wu JY, Wu SG, Yang CH (2008). Lung cancer with epidermal growth factor receptor exon 20 mutations is associated with poor gefitinib treatment response. Clin Cancer Res.

[27] Huang SF, Liu HP, Li LH (2004). High frequency of epidermal growth factor receptor mutations with complex patterns in non-small cell lung cancers related to gefitinib responsiveness in Taiwan. Clin Cancer Res.

[28] Lai RS, Xie L, Shen LS (2006). Epithelial growth factor receptor (EGFR) exon double-sequencing analysis in NSCLC. Zhonghua Zhong Liu Za Zhi.

[29] Yang TY, Tsai CR, Chen KC (2011). Good response to gefitinib in a lung adenocarcinoma harboring a heterozygous complex mutation of L833V and H835L in epidermal growth factor receptor gene. J Clin Oncol.

[30] Peng L, Song ZG, Jiao SC (2014). Efficacy analysis of tyrosine kinase inhibitors on rare non-small cell lung cancer patients harboring complex EGFR mutations. Sci Rep.

[31] Kim YC, Kim KS, Oh IJ (2012). SNP Q787Q of *EGFR* gene and efficacy of EGFR-TKI in patients with non-small cell lung cancer. Clin Cancer Res.

